# Prognostic Value of Trop‐2 Expression in Nonmetastatic Triple‐Negative Breast Cancer and Correlation With Emerging Biomarkers

**DOI:** 10.1002/cam4.70615

**Published:** 2025-03-10

**Authors:** William Jacot, Marie‐Christine Chateau, Simon Thezenas, Séverine Guiu, Nelly Firmin, Virginie Lafont, Gwendal Lazennec, Charles Theillet, Florence Boissière‐Michot

**Affiliations:** ^1^ Department of Medical Oncology Montpellier Cancer Institute Val d'Aurelle Montpellier France; ^2^ Translational Research Unit Montpellier Cancer Institute Val d'Aurelle Montpellier France; ^3^ Montpellier University Montpellier France; ^4^ Institut de Recherche en Cancérologie de Montpellier (IRCM) Montpellier France; ^5^ Biometrics Unit, Institut du Cancer Montpellier (ICM) Université de Montpellier Montpellier France; ^6^ Institut du Cancer Montpellier (ICM) Université de Montpellier Montpellier France; ^7^ CNRS UMR9005, Sys2Diag, Alcen Montpellier France

**Keywords:** basal‐like, expression, molecular apocrine, prognosis, triple‐negative breast cancer, Trop‐2

## Abstract

**Introduction:**

Triple‐Negative Breast Cancer (TNBC) is an aggressive breast cancer subtype, in which targeting the Trophoblast cell‐surface antigen‐2 (Trop‐2), using antibody‐drug conjugates (ADC), results in significant clinical improvement. However, clinicopathological correlations with Trop‐2 protein expression levels remain limited in TNBC patients.

**Methods:**

Here we assessed by immunohistochemistry (IHC) using the mouse monoclonal anti‐Trop‐2 antibody (Enzo, Cat. ENZ‐ABS380) cell membrane Trop‐2 expression levels and classified them in 3 H‐Score classes, low (< 100), moderate (100–200), and strong (> 200). We also evaluated potential associations with clinicopathological variables including basal‐like and molecular apocrine phenotypes, immune infiltrate characteristics, *PTEN* and *PIK3CA* alterations in a large retrospective series of 228 nonmetastatic TNBC patients.

**Results:**

Trop‐2 expression was evaluated as low, moderate and strong in 12.3%, 28.9%, and 58.8% of the cases respectively. Only 3 tumors showed no Trop‐2 expression. Interestingly, Trop‐2 expression was not associated with classical breast cancer clinicopathological variables, HER2 levels or molecular subtype, neither did we observe an association with relapse‐free survival. Only a marginal association with pT1 tumors was observed, which tended to express increased levels of Trop‐2 protein.

In order to determine possible fluctuations of Trop‐2 protein expression levels during the course of the disease, we studied a second independent cohort of 18 TNBC comprised of serial tissue samples (diagnostic biopsies, surgical resection specimens and corresponding patients‐derived xenografts (PDX)). Trop‐2 levels remained globally stable between cognate tumor samples with only one exception corresponding to a Trop‐2‐negative tumor giving rise to a Trop‐2‐positive PDX.

**Conclusions:**

As Trop‐2 expression appears nearly constant and independent of classical TNBC variables and outcome, association of anti‐Trop‐2 therapies with other targeted therapies can be evaluated without reducing the population in specific TNBC subgroups.

## Graphical abstract

Because Trop‐2 ADC targeting is highly promising in TNBC, we aimed at clarifying Trop‐2 expression clinicopathological correlations. Trop‐2 protein expression appeared nearly constant in TNBC, independent of classical risk variables and outcome. This is favorable to multiple therapeutic associations of anti‐Trop‐2 ADC with other drugs and avoids splintering the patient population in subclasses.

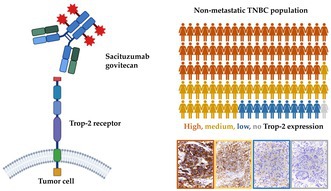

## 1 Introduction

Triple‐negative breast cancers (TNBC), defined by the lack estrogen (ER) and progesterone receptor (PR) expression, as well as no Human Epidermal Growth Factor Receptor 2 (HER2) overexpression/amplification, represent 15% of breast cancer overall [1, 2]. TNBC bear the worst prognosis of all breast cancers and despite their initial chemosensitivity show early relapse and frequent metastasis [1, 3]. Furthermore, therapeutic options remain limited in TNBC, making it an unmet medical need [4, 5, 6].

Trophoblast cell‐surface antigen‐2 (Trop‐2), encoded by the *TACSTD‐2* (tumor‐associated calcium signal transducer 2) gene, is a 40‐kDa transmembrane glycoprotein belonging to the epithelial cell adhesion molecule (EpCAM) family implicated in intracellular calcium signaling. Trop‐2 interacts with multiple regulators and transcription factors, including PI3K/PTEN/AKT and MAP/ERK pathways, in a cell context‐dependent way [7]. It plays important roles during embryonal and fetal development, and is considered a robust marker of adult stem cells. It is involved in numerous intracellular signaling pathways in normal cells [8]. Although Trop‐2 expression was originally described in trophoblasts, it has been subsequently described in an array of normal epithelial and mesenchymal tissues [7], where it is considered to regulate stem cell proliferation, migration and regenerative potential in pathological condition [7]. Trop‐2 expression has also been described in nearly all epithelial cancer types, high levels of expression being often associated with a worse prognosis [7]. However, even if it is widely expressed in human tumors, TNBC have been proposed to show higher expression levels [9].

Trop‐2 has recently become a therapeutic target of choice. Indeed, recently developed anti‐Trop‐2 antibodies conjugated with a cytotoxic agent (Antibody‐Drug Conjugates or ADC) have shown encouraging efficacy in metastatic TNBC patients. Sacituzumab govitecan, a Trop‐2‐directed monoclonal antibody coupled to a topoisomerase 1 inhibitor payload, has since been approved and is currently used in clinical practice in heavily pretreated patients with metastatic TNBC [10, 11]. Further ADCs, such as datopotamab deruxtecan, are currently under clinical development in various advanced solid tumors including non‐small cell lung cancer, digestive, gynecologic, and breast cancers [12].

Despite these clinical developments, the impact of Trop‐2 expression on TNBC biology and associations with clinicopathological and clinical outcome appear poorly investigated. Indeed, the published series are frequently investigating heterogeneous breast cancer populations and do not present findings specific of the TNBC subgroup [8, 9, 13, 14, 15].

Based on previous examples of biomarkers with diverse and sometimes complex prognostic significance, we postulated that considering the predictive value of a biomarker without considering its prognostic value could be potentially detrimental to the development of adapted therapies. Indeed, ER and HER2 bear opposite prognostic values, while being highly predictive of drug efficacy for endocrine therapy in ER+ tumors and anti‐HER2 targeted therapies in HER2 overexpressed/amplified tumors, respectively. Furthermore, it took extensive investigations to determine the actual impact of *PIK3CA* mutations in endocrine resistance supporting the clinical development of PI3K inhibitors.

The present work was undertaken to determine the potential prognostic impact of Trop‐2 expression levels in a well‐characterized cohort of 228 early‐stage TNBC patients treated at our institution. We report here the associations of Trop‐2 expression with clinicopathological features and relapse‐free survival (RFS). In addition, we examined the changes in Trop‐2 expression levels during the course of the disease, and between patient derived xenografts (PDX) [16] and their tumor of origin, in order to evaluate their suitability in preclinical studies targeting this biomarker.

## 2 Materials and Methods

### 2.1 Patients

TNBC samples were retrospectively collected from our Pathology Department (Biobank number BB‐0033–00059). Chemotherapy‐naïve, unifocal, unilateral and nonmetastatic TNBC (ER and PR < 10% and HER2 0, 1+ by immunohistochemistry (IHC) or 2+ by IHC and *ERBB2* non‐amplified by in situ hybridization (ISH)) surgically removed between 2002 and 2012 were selected. Each patient was managed in accordance with our institution's guidelines [17]. Representative tumor areas were arrayed as two cores of 1 mm in diameter in six tissue microarray (TMA). As these TMA were previously used for other projects [18, 19, 20], 228 specimens from the 296 initially punched were assessable for Trop‐2 expression. Their clinicopathological characteristics are summarized in Table 1. This study was approved by the ICM Institutional Review Board (ID number ICM‐CORT‐2022‐16) and followed the ethical principles for medical research involving human subjects (WMA Declaration of Helsinki). All patients signed a written, informed consent. This study aiming at biological markers' prognostic impact, this manuscript adheres to the REMARK guidelines.

**TABLE 1:** Patients and tumors characteristics and correlations with Trop‐2 expression.

	Low	Medium	High	Total	*p*	Low/Med	High	Total	*p*
	*n* = 28 (%)	*n* = 66 (%)	*n* = 134 (%)	*n* = 228 (%)		*n* = 94 (%)	*n* = 134 (%)	*n* = 228 (%)	
Age					0.12				0.19
< 58.4	12 (10.6%)	39 (34.5%)	62 (54.9%)	113		51 (45.1%)	62 (54.9%)	113	
≥ 58.4	16 (14.3%)	25 (22.3%)	71 (63.4%)	112		41 (36.6%)	71 (63.4%)	112	
**pT stage**					**0.002**				**0.0004**
**T1**	**8 (8%)**	**22 (22%)**	**70 (70%)**	**100**		**30 (30%)**	**70 (70%)**	**100**	
**T2**	**16 (14.2%)**	**36 (31.9%)**	**61 (54%)**	**113**		**52 (46%)**	**61 (54%)**	**113**	
**T3/T4**	**4 (26.7%)**	**8 (53.3%)**	**3 (20%)**	**15**		**12 (80%)**	**3 (20%)**	**15**	
pN stage (one Nx)					0.24				0.13
N−	21 (14.6%)	44 (30.6%)	79 (54.9%)	144		65 (45.1%)	79 (54.9%)	144	
N+	7 (8.4%)	22 (26.5%)	54 (65.1%)	83		29 (34.9%)	54 (65.1%)	83	
SBR Grade (3 Miss)					0.9				0.78
I/II	5 (9.8%)	15 (29.4%)	31 (60.8%)	51		20 (39.2%)	31 (60.8%)	51	
III	21 (12.1%)	51 (29.3%)	102 (58.6%)	174		72 (41.4%)	102 (58.6%)	174	
Histology					0.38				0.32
Ductal	7 (18.9%)	11 (29.7%)	19 (51.4%)	37		18 (48.6%)	19 (51.4%)	37	
Lobular/Other	21 (11%)	55 (28.8%)	115 (60.2%)	191		76 (39.8%)	115 (60.2%)	191	
*PIK3CA* (109 Miss)					0.43				0.71
Yes	1 (5.9%)	7 (41.2%)	9 (52.9%)	17		8 (47.1%)	9 (52.9%)	17	
No	15 (14.7%)	28 (27.5%)	59 (57.8%)	102		43 (42.2%)	59 (57.8%)	102	
*PTEN* (111 Miss)					0.17				0.06
Norm/Ampl.	11 (12.5%)	23 (26.1%)	54 (61.4%)	88		34 (38.6%)	54 (61.4%)	88	
Deletion	5 (17.2%)	12 (41.4%)	12 (41.4%)	29		17 (58.6%)	12 (41.4%)	29	
Basal‐like (2 Miss)					0.42				0.49
Yes	7 (9.7%)	25 (34.7%)	40 (55.6%)	72		32 (44.4%)	40 (55.6%)	72	
No	20 (13%)	41 (26.6%)	93 (60.4%)	154		61 (39.6%)	93 (60.4%)	154	
Mol apoc (15 Miss)					0.5				0.26
Yes	8 (9.5%)	22 (26.2%)	54 (64.3%)	84		30 (35.7%)	54 (64.3%)	84	
No	17 (13.2%)	39 (30.2%)	73 (56.6%)	129		56 (43.4%)	73 (56.6%)	129	
HER2					1				1
0	24 (12.6%)	55 (28.8%)	112 (58.6%)	191		79 (41.4%)	112 (58.6%)	191	
1+	3 (10%)	9 (30%)	18 (60%)	30		12 (40%)	18 (60%)	30	
2+	1 (14.3%)	2 (28.6%)	4 (57.1%)	7		3 (42.9%)	4 (57.1%)	7	
TCRγδ (6 Miss)					0.12				0.19
Low	12 (10.6%)	39 (34.5%)	62 (54.9%)	113		51 (45.1%)	62 (54.9%)	113	
High	16 (14.3%)	25 (22.3%)	71 (63.4%)	112		41 (36.6%)	71 (63.4%)	112	
CXCR2 (8 Miss)					0.08				0.08
Low	19 (17%)	33 (29.5%)	60 (53.6%)	112		52 (46.4%)	60 (53.6%)	112	
High	9 (8%)	30 (26.8%)	73 (65.2%)	112		39 (34.8%)	73 (65.2%)	112	
PD‐L1 (20 Miss)					0.67				0.4
Low	13 (11.9%)	34 (31.2%)	62 (56.9%)	109		47 (43.1%)	62 (56.9%)	109	
High	12 (11.5%)	27 (26%)	65 (62.5%)	104		39 (37.5%)	65 (62.5%)	104	
TILs (6 Miss)					0.13				0.09
Low	16 (16.5%)	29 (29.9%)	52 (53.6%)	97		45 (46.4%)	52 (53.6%)	97	
High	11 (8.8%)	33 (26.4%)	81 (64.8%)	125		44 (35.2%)	81 (64.8%)	125	
AdjCT (1 Miss)					0.14				0.09
Yes	7 (11.9%)	23 (39%)	29 (49.2%)	59		30 (50.8%)	29 (49.2%)	59	
No	21 (12.5%)	43 (25.6%)	104 (61.9%)	168		64 (38.1%)	104 (61.9%)	168	

*Note:* Miss: Missing data; *PIK3CA*: *PIK3CA* gene mutation; *PTEN: PTEN* mutation status; Basal‐like: Basal‐like phenotype, defined by positive staining for cytokeratin 5/6 and/or EGFR (> 10% tumor cells stained in IHC); Mol apoc: Molecular apocrine phenotype defined expression of Androgen Receptor and FOXA1 expression, using a ≥ 1% positivity cut‐off (nuclear staining); TCRγδ and CXCR2 low and high categories are defined according to their median value; PD‐L1: Programmed death‐ligand 1 stromal expression dichotomized as low and high expression (< 10% or ≥ 10% of PD‐L1‐positive stromal cells); TILs: Tumor Infiltrating Lymphocytes, using a 5% threshold; AdjCT: Adjuvant chemotherapy prescription; Significant variables (*p*‐value < 0.05) are highlighted in bold.

### 2.2 PDX and Related Human Samples

Trop‐2 IHC expression was analyzed on Formalin‐fixed, paraffin‐embedded (FFPE) samples from 22 TNBC PDX models [16] and their tumors of origin. These corresponded to 5 primary chemo‐naïve tumors, 15 post‐neo‐adjuvant chemotherapy (NAC) primary tumors and 2 recurrences. All available samples related to the tumors of origin were identified from medical files and analyzed as well.

### 2.3 Immunohistochemistry

Trop‐2 expression was assessed by IHC on serial sections from the TMAs used in previous studies in which the other biomarkers used in this study have been described (Table S1).

We selected the mouse monoclonal anti‐Trop‐2 antibody (Enzo, Cat. ENZ‐ABS380), previously used in the ancillary study of the phase III clinical trial assessing the efficacy of sacituzumab govitecan in TNBC [21]. This antibody is human‐specific and does not cross‐react with the murine form of Trop‐2. IHC was performed on 3‐μm thin TMA sections following simultaneous deparaffinization, rehydration and epitopes unmasking in low pH target retrieval solution (Dako Agilent Flex K8005). After inhibition of the endogenous peroxidase (Dako Agilent Flex Kit K8000), the slides were incubated for 10 min in a protein block solution (Dako Agilent X0909) to reduce non‐specific background. Primary antibody was used at 1:2000 dilution, incubated for 30 min at room temperature, and revealed with the Flex/Horse Radish Peroxidase (HRP) reagent (Dako Agilent Flex Kit K8000) followed by incubation with 3,3′‐diaminobenzidine (DAB) as chromogen. Finally, sections were stained with hematoxylin (Dako Agilent Ref K8008), dehydrated and mounted with permanent mounting medium. All slides were stained as a single batch to reduce experimental variability.

For PDX immunostaining, to avoid spurious staining related to the use of a mouse antibody on a mixed human/mouse tissue, anti‐Trop‐2 antibody was primarily detected with a rabbit anti‐mouse Fc specific antibody (Abcam, ab133469). This complex was then highlighted with the EnVision Rabbit reagent (Dako Agilent, K400311), a dextran polymer coupled to anti‐rabbit immunoglobulins and to several HRP molecules. DAB reagent was used as chromogen. PDX‐matched human samples were also treated with this protocol and run simultaneously to allow comparison of Trop‐2 expression levels.

Trop‐2‐immunostained TMA slides were digitalized on a Nanozoomer scanner and analyzed independently by two investigators (FBM, MCC), blinded to the clinicopathological features. Tissue microarray cores that were missing or contained fewer than 10 cancer cells or demonstrated significant artifacts were not scored. Immunostaining was scored according to the methodology described by Bardia et al. [21] to quantify the cellular membrane signal. First, we established a score grid, according to staining intensity (no labeling: 0; weak labeling: 1; moderate labeling: 2; strong labeling: 3; Figure 1A). Then, for each sampled core, the percentage of labeled invasive tumor cells in each intensity was reported. The overall cellular membrane expression was then calculated using the H‐Score method (3× % of cells with labeling intensity 3 + 2 × % of cells with intensity 2 + 1 × % of cells with intensity 1). The scores obtained ranged from 0 to 300. The agreement between the two independent analyses was high (*R* ^2^ = 0.86) and increased to a *R* ^2^ of 0.92 after consensual analysis of the 10% most discordant cases (data not shown). The mean H‐score was used for the statistical analysis.

**FIGURE 1:**
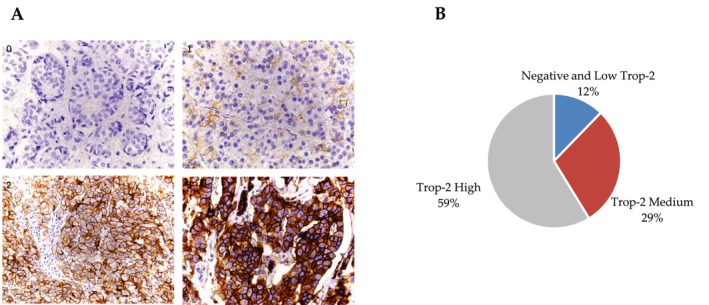
(A) Representative images of Trop‐2 immunolabeling in TNBC showing 4 typical Trop‐2 expression levels: 0: Absent (H score 0), 1: Low (< 100), 2: Medium (100–200), 3: High (> 200). Immunoperoxydase staining, ×400 magnification. (B) Immunohistochemical determination of Trop‐2 protein expression levels in our TNBC collection.

### 2.4 Molecular Analyses

Data related to *PIK3CA* and *PTEN* gene status were recovered from our previous study describing these data [22].

### 2.5 Statistical Analysis

Percentages were used to describe categorical variables, medians and ranges for continuous variables. Categorical variables were compared with the Pearson's chi‐square or Fisher's exact test. RFS was defined as the time from the surgery date to tumor recurrence. Spearman correlation coefficient was used to evaluate the level of relationship between variables. The date of the last recorded visit and the date of death were used to censor patients alive at the last follow‐up without recurrence/lost to follow‐up and patients who died without recurrence, respectively. RFS rates were calculated with the Kaplan–Meier method and compared with the log–rank test. The Cox proportional hazard model was used for multivariate analysis of all variables with a *p*‐value < 0.2 in univariate analysis. Hazard Ratio (HR) are presented with the 95% CI. STATA 16.1 (StatCorp, College Station, TX, USA) was used for all statistical analyses.

## 3 Results

### 3.1 Trop‐2 Expression

Clinicopathological characteristics of the study population were in accordance with other TNBC collections, i.e. median age 58.4 years (range: 28.5–89.1 years) with a large majority of ductal carcinoma. Treatment regimens were adjuvant chemotherapy (AdjCT) in 74% of the patients. Patients received adjuvant radiation therapy according to clinical indications.

**FIGURE 2:**
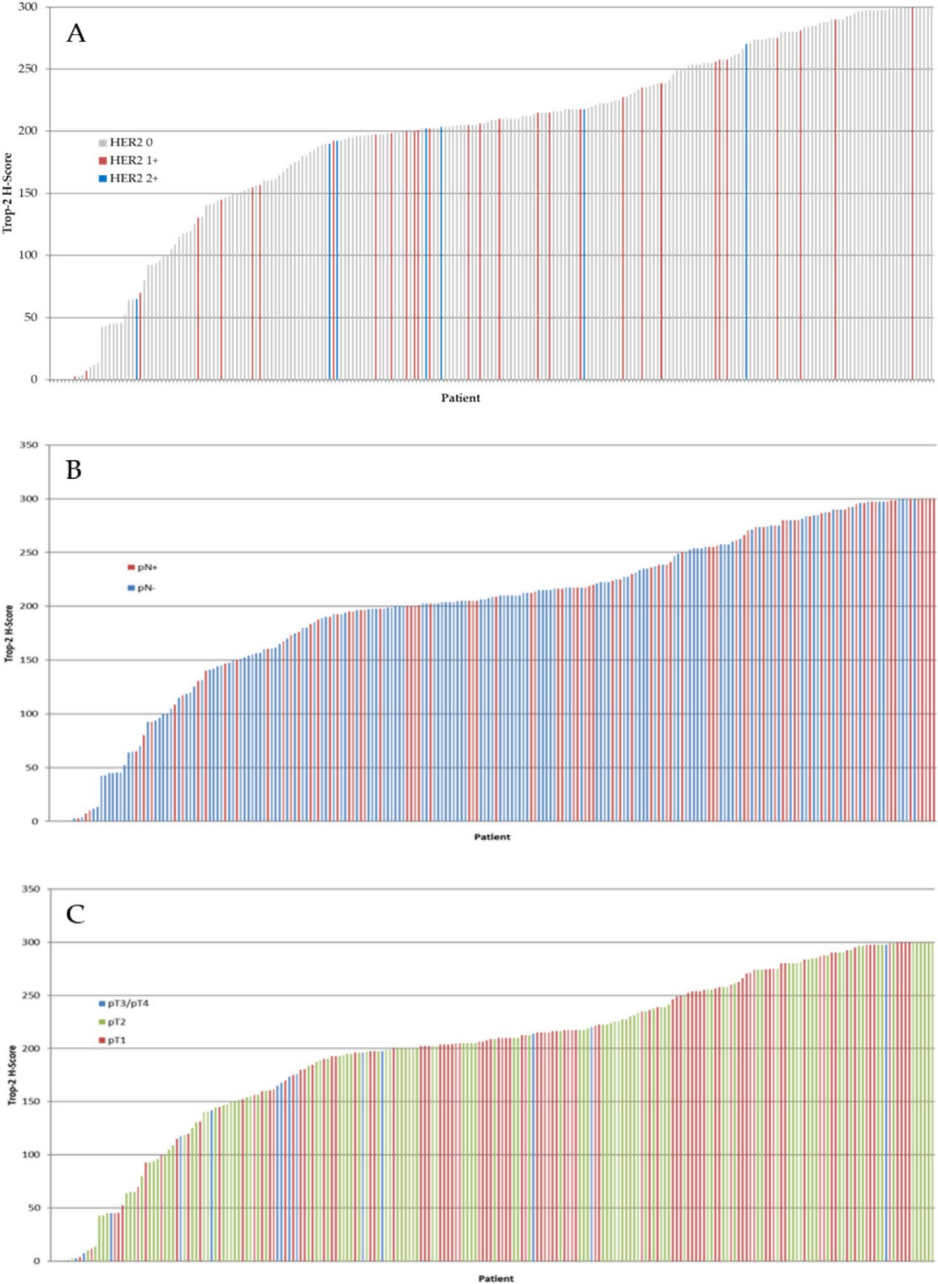
Trop‐2 expression levels (H‐Score) were evaluated according to IHC HER2 expression levels (A), pN stage (B) and pT stage (C).

Trop‐2 expression levels were assessed by IHC (Figure 1A) and tumors were classified in Trop‐2 low (< 100), medium (100–200) or high (> 200) H‐score [21], comprising 28 (12.3%), 66 (28.9%) and 134 (58.8%) of the cases respectively (Figure 1B). To gain statistical power, we distributed the tumors into two discrete groups: low and medium vs high expression. Overall, no statistical correlation was found between classical prognostic factors, nor emergent biomarkers previously evaluated in this cohort (Table 1), including HER2 levels (Figure 2A), or pN status (Figure 2B). Unexpectedly, we found a significant association with T1 stage (*p* = 0.0004), indicating that smaller tumors showed increased Trop‐2 protein expression levels (Figure 2C).

### 3.2 Survival Analyses

**FIGURE 3:**
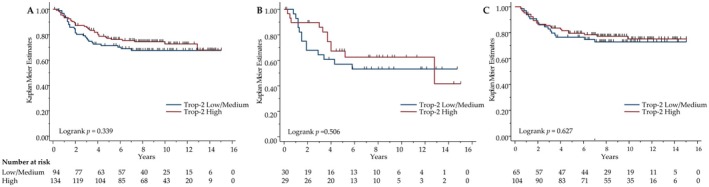
Relapse‐free survival according Trop‐2 expression level, (A) medium/Low expression levels vs High expression level, (B) in the cohort of patients without AdjCT, (C) in the cohort of patients with AdCT.

The median follow‐up in our 228 TNBC cohort was 9.8 years (95% CI [9.1; 10.5]), with 75 deaths and 64 relapses recorded. The 5‐year Overall Survival (OS) was 78.5% (95% CI [72.5–83.3]), and the 5‐year Relapse‐Free Survival (RFS) was 74.4% (95% CI [68.1–79.6]). Due to significant numbers of non‐cancer‐related deaths, we chose RFS as the relevant cancer‐associated prognostic parameter. No significant association between Trop‐2 and RFS was found (HR = 0.788, 95% CI [0.482–1.287], *p* = 0.34), whereas other parameters known to bear prognostic significance were strongly correlated to RFS (Table S2). Namely, high pT value, lymph node positivity, PTEN deletion, low (< 10%) stromal PD‐L1 expression, low (< 5%) TILs infiltration and no adjuvant chemotherapy were associated with a worse prognosis. Moreover, RFS was not significantly different in low/medium Trop‐2 vs. high Trop‐2 expressing tumors, whether or not the population received adjuvant chemotherapy, indicating an absence of predictive or prognostic impact of Trop‐2 expression (Figure 3). The results were consistent using the 3 H‐score levels (i.e., low, medium, and high Trop‐2 expression).

In the multivariate analysis (Table S3A, 107 patients included) where T and N stages, *PTEN* status, PD‐L1 stromal staining, TCRγδ infiltration, CXCR2 expression and AdjCT were significant determinants of RFS, Trop‐2 expression levels were not retained in the model. Because of elevated *PTEN* missing cases, we excluded *PTEN* in a second multivariate analysis (Table S3B, 201 patients included), in which T and N stages, PD‐L1 stromal staining, TILs levels and AdjCT were the only significant determinants of RFS.

### 3.3 Temporal Evolution and Corresponding PDX Trop‐2 Expression Profiles

**FIGURE 4:**
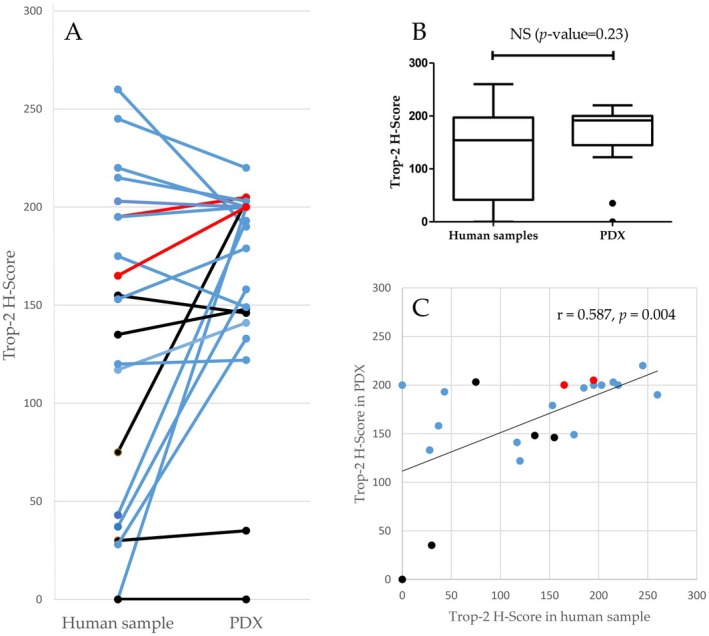
Trop‐2 expression profiles are conserved between patient tumors and corresponding PDX models; (A) Trop‐2 expression was assessed by IHC in 22 pairs of patient tumors and corresponding PDX. (B) Comparison of Trop‐2 expression between human samples and PDX; (C) Correlation of Trop‐2 expression between human samples and paired PDX. Black: Primary, chemonaïve tumors, red: Recurrent or metastatic clinical sample, blue: Post‐NAC tumors.

We analyzed 22 TNBC‐PDX models (5 chemotherapy‐naive tumors, 15 tumors removed after Neoajuvant chemotherapy (NAC) and 2 recurrences) paired with their tumors of origin, to verify whether Trop‐2 expression levels changed during the course of the disease [16]. As shown in Figure 4A,B Trop‐2 expression levels were not significantly different in the PDX tumors from those observed in their tumor of origin. A significant correlation of Trop‐2 expression was seen between the human samples and their paired PDX counterparts (*r* = 0.587, *p* = 0.004, Figure 4C). A single case of discrepancy, in which the patient's tumor was Trop‐2 negative while the corresponding PDX scored Trop‐2‐positive, was observed.

**FIGURE 5:**
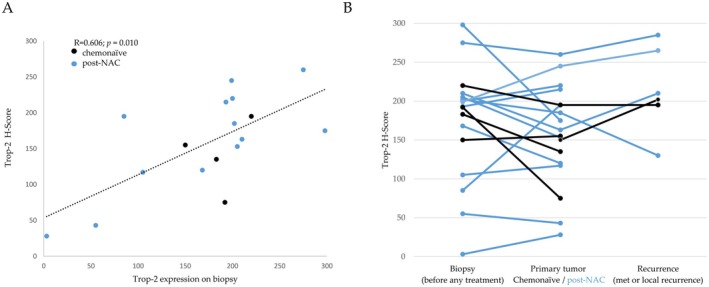
(A) Correlation analysis of Trop‐2 expression between biopsies and surgical specimens; (B) Trop‐2 IHC expression according to tumor progression (*n* = 18 patients) Black: No preoperative treatment, blue: NAC.

Next, we evaluated serial tumor samples (biopsy at diagnosis vs surgical sample of primary tumor vs recurrence) in this population of patients to evaluate the kinetics of Trop‐2 expression levels during clinical evolution (Figure 5). Eighteen patients, with at least 2 consecutive samples, were assessable. Overall, we observed a significant correlation of Trop‐2 expression between biopsy and paired surgical specimen, even in tumors sampled after NAC (*r* = 0.606, *p* = 0.010). Levels remained relatively constant between biopsies, surgical specimens and recurrences. There were no significant Trop‐2 expression differences between biopsies and surgical specimens (*p =* 0.418). Variations in Trop‐2 expression levels after NAC did not exceed those observed between biopsies and chemotherapy‐naive surgical specimens. This suggests that these variations were probably related to spatial heterogeneity of Trop‐2 expression, which was not taken into account in the analysis of biopsies.

## 4 Discussion

In the absence of recurrent druggable targets, sustained efforts have been made to refine TNBC taxonomy in order to discover therapeutic opportunities [4, 5, 6]. The recent validation of Trop‐2 as a therapeutic target, using ADCs such as sacituzumab govitecan [10], generated a renewed interest for this transmembrane molecule. However, the link between tumor biology and Trop‐2 expression levels remains poorly investigated in TNBC.

Clinicobiological characteristics associated with Trop‐2 expression are of clinical importance. Trop‐2 expression is known to be highly expressed in numerous normal and tumor tissues. However, Trop‐2 expression levels are higher in TNBC and inversely correlated with E‐cadherin expression, suggesting a role in epithelial‐mesenchymal transition [9]. Despite this apparent lack of specificity, sacituzumab govitecan demonstrated its clinical usefulness in the treatment of metastatic TNBC and was clinically validated [10]. Based on TNBC heterogeneity, we postulated that a refined knowledge of Trop‐2 biology could be of value in terms of treatment indications and therapeutic association opportunities in TNBC. Indeed, even if all the patients under sacituzumab govitecan derived a significant benefit compared to standard of care chemotherapy in the ASCENT study, a gradient of efficacy based on Trop‐2 expression levels was observed [21], arguing for the limited predictive value of Trop‐2 expression levels. Along similar lines, data suggested that resistance to sacituzumab govitecan may be linked to Trop‐2 expression levels and cellular localization [23].

Our evaluation of Trop‐2 expression levels in a homogeneous cohort of 228 localized TNBC after surgery without neoadjuvant treatment, revealed that Trop‐2 is expressed in nearly all the tumors and showed no association with specific TNBC biological subtypes or variables, nor relapse risk. Our patient cohort has been extensively characterized in previous publications [18, 19, 20, 22, 24], allowing for a comprehensive evaluation of the impact of Trop‐2 expression in TNBC.

*Trop‐2* expression in breast cancer was studied by Vidula et al. at the mRNA level using microarray data available in the I‐SPY 1, METABRIC, and TCGA datasets [25]. Although *Trop‐2* gene expression was detectable in all breast cancer subtypes, it was lower in HER2+ compared to HR+/HER2‐ and TNBC tumors and, interestingly, grade I tumors showed increased expression, possibly paralleling with our finding of accrued Trop‐2 in T1 tumor stage. Noticeably, in this study encompassing all breast cancer subtypes, *Trop‐2* gene expression was not associated with chemotherapy response or recurrence‐free survival. Similar observations were made in an IHC evaluation of Trop‐2 protein levels in a TMA comprising 404 breast tumors, including 32 TNBC [23]. Interestingly, Trop‐2 expression levels were significantly higher in TNBC patients (59.38%) compared with other subtypes (38.28%, *p* = 0.024), but no association was found with survival [23]. In further agreement with our findings, Ambrogi and coworkers, analyzing a large population comprising all breast cancer subtypes, did not observe any correlation between Trop‐2 expression and relapse [15]. However, they reported an association with overall survival, which may be misleading, since the cause of death may involve non‐cancer related causes, contralateral or other cancer sites. Moreover, the TNBC cases were not specifically characterized in their data, as the tumors were solely stratified on ER, PR, and HER2 expression levels, limiting the possibilities to extrapolate the results in the TNBC population [15]. Recently, Izci et al. [13] studied Trop‐2 protein expression in a large series of 685 stage I‐III TNBC patients and did not observe any association with either relapse or overall survival, but found a correlation with lymphovascular invasion and lymph node involvement. However, their results based on the Abcam Ab227689 monoclonal antibody, showed Trop‐2 expression patterns that differed substantially from our data. Their H‐score distribution was of 16.5%, 25.3%, and 58.2% for high, medium and low H‐scores respectively, in contrast to our findings (similar to those reported in the ASCENT trial [21]) showing 58.8%, 28.9% and 12.3% in these H‐score groups. Furthermore, they report a significant association between Trop‐2 and androgen receptor (AR) expression, which could reflect the globally small number of high‐level AR expressing tumors and could merit the use of alternative analytical methods. Indeed, using FOXA1 co‐expression evaluation to identify tumors with activated AR tumors, we did not find any correlation between the molecular apocrine profile and Trop‐2 levels of expression.

Our data show that in early TNBC, apart from a significant association with pT1 tumor size, Trop‐2 expression appeared independent of classical prognostic or predictive variables in TNBC and was not associated with survival. This is in accordance with Jeon and coworkers, [14] who only report a significant association with prognosis in the metastatic cohort. To our knowledge, we are the first to evaluate correlations of Trop‐2 expression levels with HER2‐low status, basal‐like phenotype, *PIK3CA* mutations, PTEN alterations and emerging immune infiltrate biomarkers such as PD‐L1, CXCR2 and TCRγδ. These informations could be of importance to define future strategies of therapeutic associations in TNBC. Finally, our data highlight the stability of Trop‐2 expression patterns during TNBC natural history, and between tumors of origin and corresponding PDX. These results pave the way for the study of resistance mechanisms to anti‐Trop‐2 targeted agents in these preclinical models, that encompass the full complexity of the clinical natural history of breast cancer.

Altogether, our data underline the nearly constant expression of Trop‐2 in TNBC tissues, whatever the clinicopathological variables and biological subtypes. More specifically, its expression level is unaffected by HER2 expression level, the presence of basal‐like or molecular apocrine markers, expression of emergent immune infiltrate biomarkers or presence of *PIK3CA* or *PTEN* alterations. This homogeneous expression of Trop‐2 appears of interest in the design of therapeutic associations, because it reduces the risk of segmentation of the targeted population in small size niches in the TNBC population characterized by an elevated heterogeneity. Indeed, the HER2‐low breast cancer subtype is currently targetable by trastuzumab deruxtecan [26], and basal‐like and molecular apocrine tumors are extensively investigated for sensitivity to DNA‐damaging agents (basal‐like tumors) and anti‐androgen agents (molecular apocrine tumors). Finally, the stable expression levels along tumor natural history is a further argument in favor of Trop‐2 targeting.

## Author Contributions

**William Jacot:** conceptualization (equal), project administration (equal), supervision (equal), writing – original draft (equal), writing – review and editing (equal). **Marie‐Christine Chateau:** writing – original draft (equal). **Simon Thezenas:** writing – original draft (equal). **Séverine Guiu:** writing – original draft (equal). **Nelly Firmin:** writing – original draft (equal). **Virginie Lafont:** writing – original draft (equal). **Gwendal Lazennec:** writing – original draft (equal). **Charles Theillet:** writing – original draft (equal). **Florence Boissière‐Michot:** writing – original draft (equal).

## Consent

In compliance with French law, patients were informed that their samples could be used for research purposes, and were given the opportunity to object.

## Conflicts of Interest

W.J.: Research funding (AstraZeneca), honoraria (AstraZeneca, Daiichi Sankyo), travel grants (AstraZeneca). S.G.: honoraria (Daiichi Sankyo). All other authors declare that they have no conflicts of interest.

## Institutional Review Board Statement

The study was conducted according to the guidelines of the Declaration of Helsinki, and approved by the Montpellier Cancer Institute Institutional Review Board (ID number ICM‐CORT‐2022‐16).

## Supporting information


**Data S1.** Supporting Information.
**Table S1.** Antibodies used for immunohistochemical analyses.
**Table S2.** Patients and tumors characteristics and correlations with Trop‐2 expression.
**Table S3.** Multivariate analyses to identify variables associated with RFS.

## Data Availability

The data presented in this study are available on reasonable request from the corresponding author.

## References

[1] R. Dent , M. Trudeau , K. I. Pritchard , et al., “Triple‐Negative Breast Cancer: Clinical Features and Patterns of Recurrence,” Clinical Cancer Research: An Official Journal of the American Association for Cancer Research 13, no. 15 (2007): 4429–4434. 17671126 10.1158/1078-0432.CCR-06-3045

[2] A. D. Elias , “Triple‐Negative Breast Cancer: A Short Review,” American Journal of Clinical Oncology 33, no. 6 (2010): 637–645. 20023571 10.1097/COC.0b013e3181b8afcf

[3] L. A. Carey , E. C. Dees , L. Sawyer , et al., “The Triple Negative Paradox: Primary Tumor Chemosensitivity of Breast Cancer Subtypes,” Clinical Cancer Research 13, no. 8 (2007): 2329–2334. 17438091 10.1158/1078-0432.CCR-06-1109

[4] B. D. Lehmann , J. A. Bauer , X. Chen , et al., “Identification of Human Triple‐Negative Breast Cancer Subtypes and Preclinical Models for Selection of Targeted Therapies,” Journal of Clinical Investigation 121, no. 7 (2011): 2750–2767. 21633166 10.1172/JCI45014 PMC3127435

[5] B. D. Lehmann , B. Jovanović , X. Chen , et al., “Refinement of Triple‐Negative Breast Cancer Molecular Subtypes: Implications for Neoadjuvant Chemotherapy Selection,” PLoS One 11, no. 6 (2016): e0157368. 27310713 10.1371/journal.pone.0157368 PMC4911051

[6] B. D. Lehmann and J. A. Pietenpol , “Identification and Use of Biomarkers in Treatment Strategies for Triple‐Negative Breast Cancer Subtypes,” Journal of Pathology 232, no. 2 (2014): 142–150. 24114677 10.1002/path.4280 PMC4090031

[7] S. Lenart , P. Lenárt , J. Šmarda , et al., “Trop2: Jack of all Trades, Master of None,” Cancers (Basel) 12, no. 11 (2020): 3328. 33187148 10.3390/cancers12113328 PMC7696911

[8] H. Lin , J. F. Huang , J. R. Qiu , et al., “Significantly Upregulated TACSTD2 and Cyclin D1 Correlate With Poor Prognosis of Invasive Ductal Breast Cancer,” Experimental and Molecular Pathology 94, no. 1 (2013): 73–78. 23031786 10.1016/j.yexmp.2012.08.004

[9] W. Zhao , X. Kuai , X. Zhou , et al., “Trop2 Is a Potential Biomarker for the Promotion of EMT in Human Breast Cancer,” Oncology Reports 40, no. 2 (2018): 759–766. 29901160 10.3892/or.2018.6496

[10] A. Bardia , S. A. Hurvitz , S. M. Tolaney , et al., “Sacituzumab Govitecan in Metastatic Triple‐Negative Breast Cancer,” New England Journal of Medicine 384, no. 16 (2021): 1529–1541. 33882206 10.1056/NEJMoa2028485

[11] A. Bardia , I. A. Mayer , L. T. Vahdat , et al., “Sacituzumab Govitecan‐Hziy in Refractory Metastatic Triple‐Negative Breast Cancer,” New England Journal of Medicine 380, no. 8 (2019): 741–751. 30786188 10.1056/NEJMoa1814213

[12] D. Okajima , S. Yasuda , T. Maejima , et al., “Datopotamab Deruxtecan, a Novel TROP2‐Directed Antibody‐Drug Conjugate, Demonstrates Potent Antitumor Activity by Efficient Drug Delivery to Tumor Cells,” Molecular Cancer Therapeutics 20, no. 12 (2021): 2329–2340. 34413126 10.1158/1535-7163.MCT-21-0206 PMC9398094

[13] H. Izci , K. Punie , L. Waumans , et al., “Correlation of TROP‐2 Expression With Clinical‐Pathological Characteristics and Outcome in Triple‐Negative Breast Cancer,” Scientific Reports 12, no. 1 (2022): 22498. 36577919 10.1038/s41598-022-27093-y PMC9797547

[14] Y. Jeon , U. Jo , J. Hong , G. Gong , and H. J. Lee , “Trophoblast Cell‐Surface Antigen 2 (TROP2) Expression in Triple‐Negative Breast Cancer,” BMC Cancer 22, no. 1 (2022): 1014. 36153494 10.1186/s12885-022-10076-7 PMC9509625

[15] F. Ambrogi , M. Fornili , P. Boracchi , et al., “Trop‐2 Is a Determinant of Breast Cancer Survival,” PLoS One 9, no. 5 (2014): e96993. 24824621 10.1371/journal.pone.0096993 PMC4019539

[16] S. du Manoir , B. Orsetti , R. Bras‐Gonçalves , et al., “Breast Tumor PDXs Are Genetically Plastic and Correspond to a Subset of Aggressive Cancers Prone to Relapse,” Molecular Oncology 8, no. 2 (2014): 431–443. 24394560 10.1016/j.molonc.2013.11.010 PMC5528550

[17] W. Jacot , M. Gutowski , D. Azria , and G. Romieu , “Adjuvant Early Breast Cancer Systemic Therapies According to Daily Used Technologies,” Critical Reviews in Oncology/Hematology 82, no. 3 (2011): 361. 22024387 10.1016/j.critrevonc.2011.09.002

[18] F. Boissiere‐Michot , W. Jacot , O. Massol , C. Mollevi , and G. Lazennec , “CXCR2 Levels Correlate With Immune Infiltration and a Better Prognosis of Triple‐Negative Breast Cancers,” Cancers (Basel) 13, no. 10 (2021): 2328. 34066060 10.3390/cancers13102328 PMC8151934

[19] S. Guiu , C. Mollevi , C. Charon‐Barra , et al., “Prognostic Value of Androgen Receptor and FOXA1 Co‐Expression in Non‐Metastatic Triple Negative Breast Cancer and Correlation With Other Biomarkers,” British Journal of Cancer 119, no. 1 (2018): 76–79. 29880907 10.1038/s41416-018-0142-6 PMC6035246

[20] W. Jacot , A. Maran‐Gonzalez , O. Massol , et al., “Prognostic Value of HER2‐Low Expression in Non‐Metastatic Triple‐Negative Breast Cancer and Correlation With Other Biomarkers,” Cancers (Basel) 13, no. 23 (2021): 6059. 34885167 10.3390/cancers13236059 PMC8656488

[21] A. Bardia , S. M. Tolaney , K. Punie , et al., “Biomarker Analyses in the Phase III ASCENT Study of Sacituzumab Govitecan Versus Chemotherapy in Patients With Metastatic Triple‐Negative Breast Cancer,” Annals of Oncology 32, no. 9 (2021): 1148–1156. 34116144 10.1016/j.annonc.2021.06.002

[22] W. Jacot , C. Mollevi , F. Fina , et al., “High EGFR Protein Expression and Exon 9 PIK3CA Mutations are Independent Prognostic Factors in Triple Negative Breast Cancers,” BMC Cancer 15 (2015): 986, 10.1186/s12885-015-1977-3. 26680641 PMC4683760

[23] X. Liu , T. Zhou , Y. Wang , et al., “TROP2 as Patient‐Tailoring but Not Prognostic Biomarker for Breast Cancer,” Oncotargets and Therapy 15 (2022): 509–520. 35535168 10.2147/OTT.S354048 PMC9078428

[24] F. Boissiere‐Michot , G. Chabab , C. Mollevi , et al., “Clinicopathological Correlates of Gammadelta T Cell Infiltration in Triple‐Negative Breast Cancer,” Cancers (Basel) 13, no. 4 (2021): 765. 33673133 10.3390/cancers13040765 PMC7918092

[25] N. Vidula , C. Yau , and H. Rugo , “Trophoblast Cell Surface Antigen 2 Gene (TACSTD2) Expression in Primary Breast Cancer,” Breast Cancer Research and Treatment 194, no. 3 (2022): 569–575. 35789445 10.1007/s10549-022-06660-x

[26] S. Modi , W. Jacot , T. Yamashita , et al., “Trastuzumab Deruxtecan in Previously Treated HER2‐Low Advanced Breast Cancer,” New England Journal of Medicine 387, no. 1 (2022): 9–20. 35665782 10.1056/NEJMoa2203690 PMC10561652

