# Supplementary material for: Prognostic Value of Trop‐2 Expression in Nonmetastatic Triple‐Negative Breast Cancer and Correlation With Emerging Biomarkers
Source: Cancer Med. 2025 Mar 10;14(5):e70615. doi: 10.1002/cam4.70615 (PMC11891490; doi:10.1002/cam4.70615)
Supplement: Supplementary file 1 — Data S1. Supporting Information. Table S1. Antibodies used for immunohistochemical analyses. Table S2. Patients and tumors characteristics and correlations with Trop‐2 expression. Table S3. Multivariate analyses to identify variables associated with RFS. [file CAM4-14-e70615-s001.docx]

**Supplementary tables**

**Supplementary Table S1**

| **Biomarker** | **Type** | **Clone / Ref**  **Catalog number** | **Purchaser**  **Location** | **Reference** |
| --- | --- | --- | --- | --- |
| Trop-2 | Mouse monoclonal | 01  ENZ-ABS380 | Enzo  Farmingdale, USA | - |
| FoxA1 | Goat polyclonal | C-20  HNF-3α/β | Santa Cruz Biotech.  Dallas, USA | Guiu *et al*., BJC 2018 [19] |
| AR | Mouse monoclonal | AR441  M3562 | Dako/Agilent  Santa Clara, USA | Guiu *et al*., BJC 2018 [19] |
| EGFR | Mouse monoclonal | 31G7  28-0005 | Invitrogen  Carlsbad, USA | Guiu *et al*., BJC 2018 [19] |
| CK-5/6 | Mouse monoclonal | 6D5/16 B4  M7237 | Dako/Agilent  Santa Clara, USA | Guiu *et al*., BJC 2018 [19] |
| HER2 | Rabbit polyclonal | A0485 Herceptest® | Dako/Agilent  Santa Clara, USA | Jacot *et al*., Cancers 2021 [20] |
| CXCR2 | Mouse monoclonal | E-2  sc-7304 | Santa Cruz Biotech.  Dallas, USA | Boissière-Michot *et al*., Cancers 2021a [18] |
| TCRγδ | Mouse monoclonal | H-41  sc-100289 | Santa Cruz Biotech.  Dallas, USA | Boissière-Michot *et al*., Cancers 2021b [24] |
| PD-L1 | Rabbit monoclonal | SP142  741-4860 | Roche Diagnostics Rotkreuz, Switzerland | Guiu *et al*., BJC 2018 [19] |

**Supplementary Table S2.** Univariate analysis of clinical-pathological variables in function of RFS.

|  | **Relapse Free Survival** | | |
| --- | --- | --- | --- |
|  | **Hazard Ratio** | ***p*-value** | **95%CI** |
| Age |  |  |  |
| < 58.4 | 1.000 |  |  |
| ≥ 58.4 | 1.453 | 0.137 | 0.888 - 2.379 |
| pT |  |  |  |
| T1 | 1.000 |  |  |
| T2 | 2.361 | **0.004** | 1.326 - 4.204 |
| T3/T4 | 7.274 | **<0.001** | 3.436 - 15.399 |
| pN |  |  |  |
| N- | 1.000 |  |  |
| N+ | 3.515 | **<0.001** | 2.129 - 5.804 |
| SBR Grade |  |  |  |
| I / II | 1.000 |  |  |
| III | 0.919 | 0.764 | 0.530 - 1.595 |
| Histology |  |  |  |
| Lobular/Other | 1.000 |  |  |
| Ductal | 1.003 | 0.992 | 0.539 - 1.869 |
| *PIK3CA* |  |  |  |
| Exon 9.20 | 1.000 |  |  |
| None | 0.573 | 0.195 | 0.246 - 1.330 |
| *PTEN* |  |  |  |
| Norm/Ampl. | 1.000 |  |  |
| Deletion | 2.135 | **0.039** | 1.039 - 4.386 |
| Basal-like |  |  |  |
| No | 1.000 |  |  |
| Yes | 0.731 | 0.221 | 0.442 - 1.207 |
| Mol apoc | |  |  |
| Yes | 1.000 |  |  |
| No | 0.620 | 0.058 | 0.378 - 1.016 |
| HER2 |  |  |  |
| 0 | 1.000 |  |  |
| 1+ | 1.012 | 0.972 | 0.509 - 2.013 |
| 2+ | 2.163 | 0.073 | 0.930 - 5.031 |
| CXCR2 |  |  |  |
| Low | 1,000 |  |  |
| High | 0,638 | 0.079 | 0.386 - 1.054 |
| TCRγδ |  |  |  |
| Low | 1.000 |  |  |
| High | 0.650 | 0.090 | 0.395 - 1.069 |
| PD-L1 | |  |  |
| Low | 1.000 |  |  |
| High | 0.537 | **0.021** | 0.317 - 0.911 |
| TILs |  |  |  |
| Low | 1.000 |  |  |
| High | 0.415 | **0.001** | 0.249 - 0.690 |
| AdjCT |  |  |  |
| No | 1.000 |  |  |
| Yes | 0.502 | **0.007** | 0.305 - 0.826 |
| Trop-2 expression levels |  |  |  |
| Low/Medium | 1.000 |  |  |
| High | 0.788 | 0.340 | 0.482 - 1.287 |

*PIK3CA*: *PIK3CA* gene mutation; *PTEN*: *PTEN* mutation status; Basal-like: Basal-like phenotype, defined by positive staining for cytokeratin 5/6 and/or EGFR (>10% tumor cells stained in IHC); Mol apoc phenotype: Molecular apocrine phenotype defined expression of Androgen Receptor and FOXA1, using a ≥ 1% positivity cut-off (nuclear staining) CXCR2 and TCRγδ low and high categories are defined according to their median value; PD-L1: Programmed death-ligand 1 stromal expression dichotomized as low and high expression when <10% or ≥10% of stromal cells expressed PD-L1, respectively, TILs: Tumor Infiltrating Lymphocytes, using a 5% threshold; AdjCT: Adjuvant chemotherapy prescription; Significant variables (*p*-value <0.05) are highlighted in bold.

**Supplementary** **Table S3.** Multivariate analyses to identify variables associated with RFS. **A**: Multivariate model including *PTEN* Status (significant variables). **B**: Multivariate model excluding *PTEN* value (significant variables).

| **A: Model 1   (including *PTEN*, n=107)** | | | | |  | | **B: Model 2 (excluding *PTEN*, n=201)** | | | |
| --- | --- | --- | --- | --- | --- | --- | --- | --- | --- | --- |
|  | HR | *p*-value | 95%CI |  | | HR | | *p*-value | 95%CI |  |
| pT |  |  |  |  | |  | |  |  |  |
| T1 | 1 |  |  |  | | 1 | |  |  |  |
| T2+ | 5.81 | 0.001 | 2.07 - 16.32 |  | | 2.13 | | 0.017 | 1.15 - 3.95 |  |
| pN |  |  |  |  | |  | |  |  |  |
| N- | 1 |  |  |  | | 1 | |  |  |  |
| N+ | 4.03 | 0.001 | 1.73 - 9.37 |  | | 3.51 | | <0.001 | 2.01 - 6.11 |  |
| *PTEN* Status |  |  |  |  | |  | |  |  |  |
| Norm/Ampl. | 1 |  |  |  | |  | |  |  |  |
| Deletion | 3.05 | 0.001 | 1.57 - 5.91 |  | |  | |  |  |  |
| PD-L1 Staining |  |  |  |  | |  | |  |  |  |
| Low | 1 |  |  |  | | 1 | |  |  |  |
| High | 0.31 | 0.007 | 0.13 - 0.72 |  | | 0.63 | | 0.089 | 0.37 - 1.07 |  |
| TILs |  |  |  |  | |  | |  |  |  |
| Low |  |  |  |  | | 1 | |  |  |  |
| High |  |  |  |  | | 0.54 | | 0.027 | 0.31 - 0.93 |  |
| TCRγδ |  |  |  |  | |  | |  |  |  |
| Low | 1 |  |  |  | |  | |  |  |  |
| High | 0.18 | 0.001 | 0.07 - 0.49 |  | |  | |  |  |  |
| CXCR2 cells / cm² |  |  |  |  | |  | |  |  |  |
| Low | 1 |  |  |  | |  | |  |  |  |
| High | 2.83 | 0.004 | 1.38 - 5.81 |  | |  | |  |  |  |
| Adjuvant CT |  |  |  |  | |  | |  |  |  |
| No | 1 |  |  |  | | 1 | |  |  |  |
| Yes | 0.51 | 0.074 | 0.25 - 1.07 |  | | 0.49 | | 0.007 | 0.29 - 0.82 |  |
